# Absence of a set of plasmid-encoded genes is predictive of reduced pathogenic potential in *Brachyspira hyodysenteriae*

**DOI:** 10.1186/s13567-014-0131-6

**Published:** 2014-12-16

**Authors:** Tom La, Nyree D Phillips, Jill R Thomson, David J Hampson

**Affiliations:** School of Veterinary and Life Sciences, Murdoch University, Murdoch, Western Australia 6150 Australia; Scottish Agricultural College Veterinary Services, Bush Estate, Penicuik, Midlothian, Scotland UK

## Abstract

The gene content of 14 strains of the intestinal spirochaete *Brachyspira hyodysenteriae* was compared using a DNA microarray. A consistent difference occurred in a block of four genes on the ~36 Kb plasmid, with these being present in six virulent strains and absent in eight strains with reduced pathogenic potential. These genes encoded a predicted radical S-adenosylmethionine domain protein, a glycosyl transferase group 1-like protein, an NAD dependant epimerase and a dTDP-4-dehydrorhamnose 2–5 epimerase: they may be involved in rhamnose biosynthesis and glycosylation. The absence of these plasmid genes in *B. hyodysenteriae* isolates is predictive of reduced pathogenic potential.

## Introduction, methods, and results

The anaerobic intestinal spirochaete *Brachyspira hyodysenteriae* infects the large intestine of grower and finisher pigs to cause swine dysentery (SD) [1]. In the field signs of SD may be masked or prevented by the use of antimicrobial agents, whilst dietary ingredients may influence the occurrence and severity of disease by creating conditions within the large intestine that inhibit or promote colonisation [2-4]. Differences in the *B. hyodysenteriae* strains also may account for variations in clinical outcomes. Lysons et al. [5] reported isolating *B. hyodysenteriae* from pigs in herds that were free of clinical SD, and three isolates did not produce disease in experimentally infected pigs, although they colonised the colonic mucosa of some animals. Jensen and Stanton [6] reported that *B. hyodysenteriae* reference strain B204 colonised more experimentally challenged pigs and was more virulent than the type strain B78^T^. Achacha et al. [7] challenged eight groups of weaner pigs with seven reference strains and one field isolate of *B. hyodysenteriae*, and categorised these as being of high or low virulence. Strain B204 was considered to be virulent, whilst strains B234 and A1 were considered to have low pathogenic potential. Thomson et al. [8] challenged weaner pigs with four *B. hyodysenteriae* isolates from herds with only mild diarrhoea or low prevalence of disease. Fewer of the pigs challenged with these strains showed clinical signs, pathological changes or reduced growth rates compared to pigs challenged with isolates from herds with more severe disease. Host species of origin also may be involved in differences in pathogenesis, as *B. hyodysenteriae* strains R1 and R358 isolated from common rheas (*Rhea americana*) with severe typhlitis failed to colonise pigs [9].

There have been few attempts to determine why isolates of *B. hyodysenteriae* vary in their pathogenic potential. Analysis of the complete genome sequence of virulent *B. hyodysenteriae* strain WA1, including its ~36 Kb plasmid, identified 314 (~12%) predicted open reading frames (ORFs) as having potential roles in the pathogenesis of SD [10]. These included “life style” genes such as those associated with motility and chemotaxis, those encoding surface proteins and enzymes involved in the synthesis of lipooligosaccharide (LOS), and a number of genes predicted to encode proteolytic enzymes and haemolysins/cytotoxins. Subsequent evidence for a role of the 36 Kb plasmid in pathogenesis was obtained when fewer pigs developed SD after experimental challenge with a *B. hyodysenteriae* strain lacking the plasmid compared to pigs challenged with a strain containing the plasmid [11]. The aim of the current study was to identify genes involved in pathogenesis by comparing the gene content of strains of *B. hyodysenteriae* with high or low pathogenic potential using a microarray analysis.

Spirochaete strains were obtained as frozen stock from the culture collections at Murdoch University and the Scottish Agricultural College. The *B. hyodysenteriae* strains used in the microarray analysis were selected to include six that have been reported in the literature to be virulent (B204, BW1, NSW5, NSW15, Q17 and Vic2) and eight that have been reported to colonise fewer pigs and/or cause milder disease (A1, B78^T^, B234, B6933, FM88.90, R301, SA2206 and VS1) [5-7,9]. These are recorded as “avirulent”, although they are not fully avirulent. Four other strains (P252/97; P257/97; P935/1/00; P44/15/00) that were considered to have low pathogenic potential based on field observations and results of experimental infection of pigs [8] subsequently were used in a PCR analysis of the plasmid genes to help confirm the findings from the microarray work.

Spirochaetes were propagated in Kunkle’s pre-reduced anaerobic broth [12], and at a density of approximately 10^8^ cells/mL they were harvested by centrifuging at 10 000 × *g*, and counted under a phase contrast microscope.

Custom GeneChips were designed and manufactured by Affymetrix (Santa Clara, California) using the predicted ORFs from *B. hyodysenteriae* WA1 [10]. A total of 1993 of the 2638 ORFs encoded on the genome and 24 of the 31 ORFs encoded on the plasmid were represented on the chip. Each ORF was represented on the array by a series of 13 different pairs of 25-mer oligonucleotide probes. Each probe pair consisted of a perfect match oligonucleotide and a mismatch oligonucleotide. The probe set was pruned against all other *B. hyodysenteriae* WA1 sequences on the chip and the Affymetrix Standard Pruning Libraries for *Escherichia coli* and *Pseudomonas* spp. genomes.

Microarray-based comparative genomic hybridisation (CGH) analysis was used to compare the gene content of the six virulent and eight “avirulent” *B. hyodysenteriae* strains with the gene content of virulent *B. hyodysenteriae* strain WA1. A single channel microarray analysis was performed using the Affymetrix GeneChips. High molecular weight DNA was extracted from the *B. hyodysenteriae* cells using the DNeasy Blood and Tissue Kit (Qiagen) according to the manufacturer’s instructions. The integrity of all DNA preparations was confirmed by PCR using species-specific primers [13]. Purified DNA was digested with *Rsa*1 and the restriction fragments were labelled with a fluorescent cyanide dye (Cy3) using the BioPrime Array CGH Genomic Labelling System (Invitrogen). The labelled genome fragments were hybridised to the *B. hyodysenteriae* GeneChip under moderately stringent conditions (37 °C) in a Hybridisation Oven 645 (Affymetrix) for 16 h. The GeneChips were washed and labelled using the GeneChip Hybridisation, Wash and Stain Kit (Affymetrix), using a Fluidics Station 450 (Affymetrix). The GeneChips were scanned using the Scanner 3000 (Affymetrix) and the hybridisation intensity data converted to numeric values using the GeneChip Operating Software (GCOS, Affymetrix). Affymetrix default software cut-off values were used and each ORF was categorized as “Present”, “Marginal” or “Absent”. *B. hyodysenteriae* strain WA1 and *B. innocens* strain 4/71 served as positive and negative controls, respectively.

For the seven plasmid-encoded genes absent from the GeneChip, three primer pairs were designed for PCR amplification of each (Table 1). For the four genes present in the plasmid of the virulent strains and absent in the “avirulent” strains (BHWA1_02678 to BHWA1_02681), three unique primer pairs were designed for the PCR amplification of each ORF (Table 2). High molecular weight DNA from all strains was subjected to PCR using HotStar*Taq* DNA Polymerase (Qiagen) according to the manufacturer’s instructions. The annealing temperature used for each primer was set at 5 °C less than the optimal annealing temperature to allow for a moderate stringency similar to that of the microarray hybridisation. The amplification products were electrophoresed through an agarose gel, stained with ethidium bromide and viewed over ultraviolet light.

**Table 1:** **PCR primers used for the amplification of** ***B. hyodysenteriae*** **plasmid genes not on the GeneChip**

**Primer name** ^**a**^	**Sequence (5′ – 3′)**	**Product size**
BHWA1_02666a-F	gagaagaatatttaatacctttaatag	217 bp
BHWA1_02666a-R	cattcatatatataataataatggttgg	
BHWA1_02666b-F	ccaaccattattattatatatatgaatg	549 bp
BHWA1_02666b-R	ctaaatctctatattgtttaactatag	
BHWA1_02666c-F	ctatagttaaacaatatagagatttag	177 bp
BHWA1_02666c-R	ctatttttatacctaatatggtgaatat	
BHWA1_02667a-F	actggagttgctggatttataggatc	560 bp
BHWA1_02667a-R	aagtcaggtctctgtctctttcc	
BHWA1_02667b-F	caaataaagatcatactgttataggaatag	597 bp
BHWA1_02667b-R	atgtatagtcacgcatagtgg	
BHWA1_02667c-F	tgtaatacatttagcaggatatgg	384 bp
BHWA1_02667c-R	ggtataggattattttcaagtatcag	
BHWA1_02668a-F	gttcataccatttagaaaaagaagag	701 bp
BHWA1_02668a-R	gttcataccatttagaaaaagaagag	
BHWA1_02668b-F	agaacaaaacaacataaagcatc	206 bp
BHWA1_02668b-R	catcagtaaaacaaatataatccc	
BHWA1_02668c-F	cctgagcattatggactttc	240 bp
BHWA1_02668c-R	tgtactgtctgattttttatggtc	
BHWA1_02672a-F	aaatgtagaagatattgtattgcc	417 bp
BHWA1_02672a-R	acctctcctatatgttttttatacttag	
BHWA1_02672b-F	attactacaaaatgtactctaaaatgtaag	546 bp
BHWA1_02672b-R	ccatactatatgacaaaaataaaatctag	
BHWA1_02672c-F	tatctaagtataaaaaacatataggagagg	498 bp
BHWA1_02672c-R	cagcacaaaactcacatagtg	
BHWA1_02673a-F	aaatacttgtcaataatcttagtgg	1819 bp
BHWA1_02673a-R	tttcatcataagcaaaaataatatc	
BHWA1_02673b-F	gtaagtggaaaaagaatgaaacatac	1032 bp
BHWA1_02673b-R	agattgtcttgacgaataaaag	
BHWA1_02673c-F	aataaatatgacattaaaggaataaaaatc	805 bp
BHWA1_02673c-R	ctattgttagtagcaaaataataaaaatac	
BHWA1_02674a-F	atttagaagatgtaatacctttagagg	249 bp
BHWA1_02674a-R	tcattttcgctatatttttatttac	
BHWA1_02674b-F	ttatacaaaataggagagcctttag	363 bp
BHWA1_02674b-R	atcgcaataatctgaaaatg	
BHWA1_02674c-F	gtatgtacttatcttttttattctattgtc	194 bp
BHWA1_02674c-R	catattggatttttatctctatgtc	
BHWA1_02675a-F	attggatagaacatagagggag	301 bp
BHWA1_02675a-R	actgtatcatttgctatttcattag	
BHWA1_02675b-F	tataaaaactataagaatatctctacaagg	367 bp
BHWA1_02675b-R	aacatataaggtataaaatggttgag	
BHWA1_02675c-F	cctcaaccattttataccttatatg	184 bp
BHWA1_02675c-R	taactatattttctcgttttccttg	

^a^Primers named according to the plasmid gene they are designed to amplify.

**Table 2:** **PCR primers for amplification of the four genes on the plasmid that were absent in the “avirulent”** ***B. hyodysenteriae*** **strains but present in all the virulent strains**

**Primer name** ^**b**^	**Sequence (5′ – 3′)**	**Product size**
BHWA1_02678a-F	tagaaacggtaatcccattag	322 bp
BHWA1_02678a-R	taaaagcgaagcattagtagtag	
BHWA1_02678b-F	tgataaagtaaatagggtagatactactac	420 bp
BHWA1_02678b-R	aaatacataaggacaaaccattac	
BHWA1_02678c-F	ttttgctgaaatggtagagtatg	558 bp
BHWA1_02678c-R	tatagtttttactgattctttattgacatc	
BHWA1_02679a-F	ttggtggaggagttggtac	237 bp
BHWA1_02679a-R	tgataaagaagaggatgattcc	
BHWA1_02679b-F	tcaggtataaatccgcctaatg	495 bp
BHWA1_02679b-R	caggtactaaaccagcagtcatag	
BHWA1_02679c-F	ggttttatatgtaagaatgaagatg	329 bp
BHWA1_02679c-R	cttagaatttgaagtccagtttg	
BHWA1_02680a-F	gaagtttttggcataggaac	289 bp
BHWA1_02680a-R	tcattttcttttaatggtgtatc	
BHWA1_02680b-F	aaaaggctatttagaatctgaag	280 bp
BHWA1_02680b-R	cattatctccataagtatagaaaactctac	
BHWA1_02680c-F	tggtgatatagcaaaaagtatagc	183 bp
BHWA1_02680c-R	ccctacaataattttaggttcttcag	
BHWA1_02681a-F	gtgggaaaaataaatctgaag	345 bp
BHWA1_02681a-R	gcttaattctacagtaaaatgctg	
BHWA1_02681b-F	gtaatgatgaacttaaaaaatcaggtattg	266 bp
BHWA1_02681b-R	attccatgagccacataaggag	
BHWA1_02681c-F	aaacagtcaaatatgagttatagtgc	391 bp
BHWA1_02681c-R	aaatctggtatacttttatccttttc	

^b^Primers named according to the plasmid gene they are designed to amplify.

For the four strains that were not hybridised on the microarray, spirochaetes were harvested from 1 mL of culture by centrifugation and the pellet was resuspended 1 mL sterile TE buffer. The resuspended cells were boiled for 2 min, the cellular debris pelleted by centrifugation and the supernatant subjected to PCR.

The categorised values for the CGH analysis and the PCR results for the genes missing from the microarray were merged. Genes which were present or marginal in the virulent strains but absent in the low-virulence strains were identified. None of the plasmid genes gave marginal signals.

In CGH analysis *B. innocens* 4/71 shared only 328 of the 1993 WA1 genes, whilst the six virulent *B. hyodysenteriae* strains shared between 1919 and 1982 (96.3 to 99.4%) of the genes and 1871 (93.9%) genes were common to all virulent strains (ie were core genes). The eight “avirulent” strains shared 1874 to 1930 (90.4 to 96.8%) of the WA1 genes, of which 1757 (88.2%) were common to all the “avirulent” strains. All 14 strains shared 1741 (87.4%) of the WA1 genes. Only five genes (of unknown function) were specific to *B. hyodysenteriae* WA1 (BHWA1_01317, BHWA1_00005, BHWA1_00006, BHWA1_01358 and BHWA1_00574). The data from the CGH analysis have been deposited at the Gene Expression Omnibus public repository [14].

The CGH analysis identified four genes that were present in all six virulent strains (and WA1) and absent in all eight “avirulent” strains. These were located as a block of adjacent genes on the 36 Kb plasmid (positions 13–16), and were predicted to encode a radical S-adenosylmethionine (SAM) protein, a glycosyltranserase, an NAD-dependent epimerase and an dTDP-4-dehydrorhamnose 3,5-epimerase (*rfb*C), which are all catalytic enzymes potentially involved in LOS biosynthesis and/or glycosylation (Table 3). Two adjacent plasmid genes (positions 11 and 12) predicted to encode radical SAM domain proteins also were absent from the eight “avirulent” strains and were present in five of the six virulent strains tested (absent in NSW15). “Avirulent” strains A1 and FM88.90 did not possess the plasmid genes.

**Table 3:** **Distribution of the** ***B. hyodysenteriae*** **plasmid genes amongst the virulent and “avirulent” strains determined by GeneChip microarray and PCR**

**Gene (position on plasmid)**	**Putative function**	**Virulent strains**						**Avirulent strains**							
		**B204**	**BW1**	**NSW5**	**NSW15**	**Q17**	**Vic 2**	**A1**	**B78**	**B234**	**B6933**	**FM88.90**	**R301**	**SA2206**	**VS1**
**BHWA1_02666 (1)**	glycosyltransferase	P	P	P	P	P	P	A	P	P	P	A	P	P	P
**BHWA1_02667 (2)**	NAD dependent epimerase/dehydratase	P	P	P	P	P	P	A	P	P	P	A	P	P	P
**BHWA1_02668 (3)**	glycosyl transferase, family 2	P	P	P	P	P	P	A	P	P	P	A	P	P	P
BHWA1_02669 (4)	glycosyl transferase, group 1-like protein	P	P	P	P	P	P	A	P	P	P	A	P	P	P
BHWA1_02670 (5)	glycosyl transferase, group 1	P	P	P	P	P	P	A	P	P	P	A	P	P	P
BHWA1_02671 (6)	glycosyl transferase	P	P	P	P	P	P	A	P	P	P	A	P	P	P
**BHWA1_02672 (7)**	hydrolase (HAD superfamily) protein	P	P	P	P	P	P	P	P	P	P	A	P	P	P
**BHWA1_02673 (8)**	hydrolase (HAD superfamily) protein	P	P	P	P	P	P	P	P	P	P	A	P	P	P
**BHWA1_02674 (9)**	Radical SAM domain protein	P	P	P	P	P	P	P	P	P	P	A	P	P	P
**BHWA1_02675 (10)**	Radical SAM domain protein	P	P	P	P	P	P	P	P	P	P	A	P	P	P
BHWA1_02676 (11)	Radical SAM domain protein	P	P	P	A	P	P	A	A	A	A	A	A	A	A
BHWA1_02677 (12)	Radical SAM domain protein	P	P	P	A	P	P	A	A	A	A	A	A	A	A
*BHWA1_02678 (13)*	*Radical SAM domain protein*	*P*	*P*	*P*	*P*	*P*	*P*	*A*	*A*	*A*	*A*	*A*	*A*	*A*	*A*
*BHWA1_02679 (14)*	*glycosyl transferase, group 1-like protein*	*P*	*P*	*P*	*P*	*P*	*P*	*A*	*A*	*A*	*A*	*A*	*A*	*A*	*A*
*BHWA1_02680 (15)*	*NAD dependent epimerase*	*P*	*P*	*P*	*P*	*P*	*P*	*A*	*A*	*A*	*A*	*A*	*A*	*A*	*A*
*BHWA1_02681 (16)*	*dTDP-4-dehydrorhamnose 3,5-epimerase (* *rfb*C)	*P*	*P*	*P*	*P*	*P*	*P*	*A*	*A*	*A*	*A*	*A*	*A*	*A*	*A*
BHWA1_02682 (17)	Radical SAM domain protein	P	P	P	P	P	P	A	P	P	P	A	P	P	P
BHWA1_02683 (18)	Glucose-1-phosphate cytidylyltransferase (*rfb*F)	P	P	P	P	P	P	A	P	P	P	A	P	P	P
BHWA1_02684 (19)	plasmid partitioning protein (*cds*M)	P	P	P	P	P	P	A	P	P	P	A	P	P	P
BHWA1_02685 (20)	hypothetical protein	P	P	P	P	P	P	A	P	P	P	A	P	P	P
BHWA1_02686 (21)	replicative DNA helicase	P	P	P	P	P	P	A	P	P	P	A	P	P	P
BHWA1_02687 (22)	DNA primase-like protein	P	P	P	P	P	P	A	P	P	P	A	P	P	P
BHWA1_02688 (23)	integrase	P	P	P	P	P	P	A	P	P	P	A	P	P	P
BHWA1_02689 (24)	alpha-1,2-fucosyltransferase	P	A	A	P	A	P	A	P	P	P	A	A	P	P
BHWA1_02690 (25)	lipopolysaccharide biosynthesis protein-like protein	P	P	P	P	P	P	A	P	P	P	A	P	P	P
BHWA1_02691 (26)	dTDP-D-glucose 4,6-dehydratase (*rfb*B)	P	P	P	P	P	P	A	P	P	P	A	P	P	P
BHWA1_02692 (27)	glucose-1-phosphate thymidylyltransferase (*rfb*A)	P	P	P	P	P	P	A	P	P	P	A	P	P	P
BHWA1_02693 (28)	dTDP-4-keto-L-rhamnose reductase (*rfb*D)	P	P	P	P	P	P	A	P	P	P	A	P	P	P
BHWA1_02694 (29)	dTDP-4-dehydrorhamnose 3,5-epimerase (*rfb*C)	P	P	P	P	P	P	A	P	P	P	A	P	P	P
BHWA1_02695 (30)	hypothetical protein	P	P	P	P	P	P	A	P	P	P	A	P	P	P
BHWA1_02696 (31)	glycosyltransferase	P	P	P	P	P	P	A	P	P	P	A	P	P	P

The names of seven of the plasmid genes that were not on the microarray (genes 1–3; 7–10) but were amplified by PCR are marked in bold. The microarray results for the four genes (genes 13–16) that were absent in the “avirulent” strains but present in the virulent strains are italicised. Genes 11 and 12 also were absent in the “avirulent” strains and were present in five of the six virulent strains (NSW15 being the exception).

P represents gene present, A represents gene absent. Strains A1 and FM88-90 lacked all 31 plasmid genes.

Other probe sets identified differences between individual virulent and “avirulent” strains, but these were not consistent. Seven sets identified individual chromosomal genes (of unknown function) in the virulent strains that were absent in six or seven of the eight “avirulent” strains (Table 4). Virulent strain NSW15 lacked four of the genes that were absent in the “avirulent” strains. There were no genes present in all the “avirulent” strains and absent in the virulent strains.

**Table 4:** **Other chromosomal genes absent in six or seven of the eight “avirulent” strains but present in five or six of the virulent strains**

		**Virulent strains**						**Avirulent strains**							
**Probe set**	**Size (base pairs)**	**B204**	**BW1**	**NSW5**	**NSW15**	**Q17**	**Vic2**	**B6933**	**B78** ^**T**^	**FM88-90**	**A1**	**B234**	**VS1**	**SA2206**	**R301**
GeneBh581_at	318	P	P	P	P	P	P	A	A	A	P	A	A	A	A
GeneBh398_at	135	P	P	P	P	P	P	A	A	P	P	A	A	A	A
GeneBh1267_at	117	P	P	P	P	P	P	A	A	P	A	A	A	A	P
GeneBh1690_at	537	P	P	P	A	P	P	A	A	A	P	A	A	A	A
GeneBh310_at	93	P	P	P	A	P	P	M	P	A	A	A	A	A	A
GeneBh551_at	819	P	P	P	A	P	P	P	A	A	A	A	A	P	A
GeneBh513_at	1176	P	P	P	A	P	P	A	A	A	A	A	A	P	P

All genes are of unknown function. A, absent; P, present; M, marginal (weak signal).

PCR analysis of the seven genes on the plasmid that were not represented on the GeneChip indicated that all the *B. hyodysenteriae* strains except A1 and FM88.90 possessed all seven genes (Table 3).

When used on the four additional strains of low pathogenic potential that were not tested on the microarray [8], the PCRs targeting plasmid-borne genes 11 – 16 failed to amplify them from strain P944/15/10, and failed to amplify the radical SAM genes (genes 10, 11 and 12) from strain P935/1/00.

## Discussion

The “avirulent” strains used in this study were not all completely avirulent, but had been shown to colonise fewer experimental animals after challenge and/or cause milder disease compared to typical field strains. They could be defined as having reduced pathogenic potential compared to typical pathogenic strains.

A striking difference in gene profiles was observed in a block of four genes located on the plasmid which were present in the virulent strains but not in these “avirulent” strains. PCR analysis showed that two adjacent genes encoding radical SAM domain proteins also were absent in the “avirulent” strains, but they were absent in one of the virulent strains as well so that the correlation was less strong.

PCR screening of four additional strains broadly supported the existence of a correlation between the plasmid genes and virulence, although two of the “avirulent” strains possessed these genes and one only lacked the radical SAM domain genes. Clearly reduced virulence may be associated with factors other than absence of the plasmid genes. The fact that the “avirulent” strains used in the study were only able to colonise and induce disease in a small proportion of pigs in previous studies suggests that the genes are most likely to be involved in facilitating aspects of colonisation rather than in lesion development. This could involve specific changes to surface molecules including such things as modification to LOS composition or glycosylation of surface proteins that are involved in attachment. Strain R301 lacked the plasmid genes and induces disease in rheas but not in pigs [9], hence suggesting that there is species-specificity in the colonisation process.

There was no evidence that other genes on the plasmid were correlated with virulence. Some other chromosomal genes of unknown function were absent in some of the avirulent strains, but the lack of consistency did not suggest that they are important in the context of pathogenesis. It is possible that other genes not represented on the microarray may be associated with virulence, and might have had a different distribution in the two sets of isolates. A more detailed understanding could be obtained by undertaking whole genomic sequencing of the *B. hyodysenteriae* strains.

The products of the plasmid genes that were present in the virulent strains but absent in the other strains apparently influence the pathogenesis of SD, although gene expression was not investigated. Neither was it possible to specifically inactivate the identified genes to determine whether this affects the phenotype and behaviour of the strains when used to infect pigs, due to lack of easily applicable means to genetically manipulate *B. hyodysenteriae* in a targeted way. Further studies of this sort should be undertaken in the future to help understand the role of the gene products.

Of the predicted gene products, radical SAM dependent enzymes catalyze a diverse assortment of reactions involved in numerous important biological pathways in other bacteria, including the biosynthesis of a wide number of enzyme cofactors and secondary metabolites, anaerobic oxidations, biosynthesis and repair of DNA, and general bacterial metabolism. SAM also is important for the production of a universal quorum-sensing signal in bacteria that regulates pathogen-host interactions. Inhibition of this enzyme in other bacteria can lead to attenuation of virulence [15].

The *rfb* genes shape the structure of the LOS O-antigens that are likely to be involved in host-defence mechanism. In most bacteria, the *rfb* genes are found on the genome as a cluster and are believed to have been acquired through lateral gene transfer [16]. An *rfb*ABCD gene cluster was present on the plasmid of all the *B. hyodysenteriae* strains tested in this study, however the additional *rfb*C gene on the plasmid of the virulent strains might contribute to virulence. Both *rfb*C genes possess the dTDP-4-dehydrorhamnose 3,5-epimerase conserved domain, but are only 52% similar at the protein level. *B. hyodysenteriae* LOS is considered to contribute to virulence as it has various biological effects in vitro and in vivo [17-21].

Glycosyl transferases play an important role in pathogenicity of most pathogenic bacteria [22-24]. They catalyse the transfer of sugar moieties to acceptor substrates such as oligosaccharides, monosaccharides, proteins and lipids. Protein glycosylation can have roles in host interactions, protection against proteolytic cleavage, protein assembly, antigenic variation, and protective immunity. Glycosyl transferases are highly specific with respect to both the donor sugar and acceptor, and to date the exact specificities of most have not been determined [25].

Overall the findings of this study indicate that a block of specific genes on the *B. hyodysenteriae* plasmid are involved in facilitating colonisation and development of disease. Isolates lacking these genes can be predicted to have a lower pathogenic potential in pigs than those that have the genes.
